# Differential mRNA Accumulation upon Early *Arabidopsis thaliana* Infection with ORMV and TMV-Cg Is Associated with Distinct Endogenous Small RNAs Level

**DOI:** 10.1371/journal.pone.0134719

**Published:** 2015-08-03

**Authors:** Diego Zavallo, Humberto Julio Debat, Gabriela Conti, Carlos Augusto Manacorda, Maria Cecilia Rodriguez, Sebastian Asurmendi

**Affiliations:** 1 Instituto de Biotecnología, CICVyA-INTA, Hurlingham, Buenos Aires, Argentina; 2 Consejo Nacional de Investigaciones Científicas y Técnicas (CONICET), Buenos Aires, Argentina; 3 Instituto de Patología Vegetal (IPAVE), Centro de Investigaciones Agropecuarias (CIAP), INTA, Córdoba, Argentina; Louisiana State University, UNITED STATES

## Abstract

Small RNAs (sRNAs) play important roles in plant development and host-pathogen interactions. Several studies have highlighted the relationship between viral infections, endogenous sRNA accumulation and transcriptional changes associated with symptoms. However, few studies have described a global analysis of endogenous sRNAs by comparing related viruses at early stages of infection, especially before viral accumulation reaches systemic tissues. An sRNA high-throughput sequencing of *Arabidopsis thaliana* leaf samples infected either with *Oilseed rape mosaic virus* (ORMV) or crucifer-infecting *Tobacco mosaic virus* (TMV-Cg) with slightly different symptomatology at two early stages of infection (2 and 4dpi) was performed. At early stages, both viral infections strongly alter the patterns of several types of endogenous sRNA species in distal tissues with no virus accumulation suggesting a systemic signaling process foregoing to virus spread. A correlation between sRNAs derived from protein coding genes and the associated mRNA transcripts was also detected, indicating that an unknown recursive mechanism is involved in a regulatory circuit encompassing this sRNA/mRNA equilibrium. This work represents the initial step in uncovering how differential accumulation of endogenous sRNAs contributes to explain the massive alteration of the transcriptome associated with plant-virus interactions.

## Introduction

One of the attention-grabbing goals in plant pathology is to understand the mechanisms involved in the production of symptoms during plant-virus interaction. Significant progress has been made in the comprehension of how viruses induce disease, how hosts develop resistance and how viruses co-opt the defense system to impair resistance, particularly through RNA silencing [1, 2]. However, it remains unclear how viruses manage to exploit their hosts successfully in order to break their defense machinery and whether symptoms are indeed a host response as a direct result of infection, a side effect of viral accumulation, or both [3].

Plant development is affected by plant–virus interactions, which interfere with a broad range of cellular processes, such as hormonal regulation, cell cycle control, endogenous transport of macromolecules [1] as well as extensive virus associated transcriptome reprogramming [4]. In recent years, small RNAs (sRNAs) have been demonstrated to play important roles in plant development and host-pathogen interaction [5–7]. Several classes of endogenous sRNAs have been described. Although these endogenous sRNAs are heterogeneous in sequence, genomic distribution, biogenesis, and action, most of these molecules mediate repressive gene regulation through RNA silencing [8].

A major distinction can be observed between small RNAs derived from single-stranded precursors with a hairpin structure (hpRNA) and those derived from double-stranded precursors (dsRNA), aberrant or repetitive RNA. Those derived from single-stranded precursors mainly originate microRNAs (miRNAs), whereas the others primarily produce short interfering RNAs (siRNAs).

MiRNAs derive from RNA polymerase II transcripts that adopt foldback structures which are recognized and processed by DICER-LIKE 1 (DCL1) for their maturation. These miRNAs precursors then give rise to ~20-24nt sRNAs that guide Argonaute 1 (AGO1) and exert as negative regulators of complementary mRNAs [9, 10]. The interaction of miRNAs with target transcripts results in either target degradation (typical of plants) or translation inhibition (typical of animals) [11, 12].

siRNAs originate from dsRNA usually by bidirectional transcription, via RNA dependent RNA polymerase (RDR) or aberrant RNA. Eventually, siRNAs guide sequence-specific transcriptional silencing mainly through DNA cytosine methylation or RNA target degradation [13]. One of the main siRNA classes is the heterochromatic siRNAs (hcsiRNAs), which are 23–24nt long, and depend mostly on RDR2 and DCL3 [13], AGO4 [14] and PolIV [15]. The secondary siRNAs constitute another class of siRNAs. The sRNA targeting of an initial primary transcript leads to RDR6 recruitment, complementary RNA strand synthesis, and phased processing of the resulting dsRNA into secondary siRNAs by DCL4 [9]. Some of the generated secondary siRNAs are capable of acting in *trans* (TasiRNAs) to direct repression of distinct mRNA targets, such as TAS transcripts [16]. Recent studies have demonstrated that secondary siRNAs are also generated from protein coding genes in many plant genomes [17–20].

Viruses induce the RNA silencing machinery in infected plants through viral dsRNA replicative forms, production of aberrant RNA, amplification of RNA transcripts by virus-encoded RDR or by complex folding of virus RNA forms [21]. This process is coordinated by DCL4, DCL2 as well as several RDRs and results in the biogenesis of viral derived small RNA species (vsRNAs) that guide viral RNA silencing in *cis* as an antiviral defense response in plants [22].

Several studies have reported the relationship between viral infections, endogenous sRNA accumulation (especially miRNAs) and transcriptional changes associated with symptoms, while others have focused on the impact of vsRNAs in both pathogenicity and symptom production [5, 23–26]. However, few studies have described a global analysis of endogenous sRNAs by comparing related viruses at early stages of infection, especially before viral accumulation reaches systemic tissues.

In this study, the sRNAs populations at early stages of tobamovirus infections in *Arabidopsis thaliana* were characterized by using high-throughput sequencing technologies that allow both qualitative discoveries of sRNA classes as well as quantitative profiling of sRNA populations. For this purpose, two strains of cruciferous-infecting tobamoviruses (90.9% genome sequence homology between both strains) to infect *A*. *thaliana* were employed: *Oilseed rape mosaic virus* (ORMV) and *Tobacco mosaic virus* strain Cg (TMV-Cg). A global analysis of the different types of endogenous sRNAs was described.

Our analysis showed that viral infection, at very early stages of infection strongly modifies the abundance of numerous types of sRNA species, despite that no virus was detected in the systemic tissues of the infected plants. This finding indicates that a signaling process precedes the accumulation of viral particles in systemic tissues. Moreover, the patterns of endogenous sRNAs derived from protein coding genes were distinctively altered in systemic tissue of virus infected plants. Based in these observations we propose that a recursive mechanism is involved in a regulatory circuit encompassing this sRNA/mRNA equilibrium, which in turn contributes in the alteration of the transcriptional landscape of virus infected plants.

## Materials and Methods

### Plant material, viral infection and sample collection

Seeds were stratified at 4°C for 3 days. *A*. *thaliana* Col-0 plants were grown under standard conditions [27] in controlled environmental chambers (22°C 16/8 h light/dark). To quantify TMV-Cg [28] and ORMV [29] inoculum, *N*. *tabacum (NN)* plants were infected from serial dilutions of viral extracts. Local lesions were counted and the inoculum stored at -80°C until infection. Mock-inoculated plants were rubbed with sodium sulfite buffer (1% K_2_HPO_4_ + 0.1% Na_2_SO_3_ [wt/vol]). *Arabidopsis* plants were mechanically inoculated in their third true leaf at stage 1.08 [27], because those leaves are almost fully developed by the time of the procedure. The systemic leaves 8 and 6, and 11 and 8 were taken at 2 and 4dpi respectively for RNA isolation. Five independent plants for each treatment were used. These leaves were snap-cooled in liquid nitrogen and stored at -80°C. For pigments measurement individual leaves #5 were taken and extracted using 96% ethanol. Absorbance was measured at 664 and 649 nm in a Multiskan Spectrum (ThermoFisher Scientific, Massachusetts, USA). To study the differences in severity between strains, phenotypic measurements were performed. The impact of virus infection on plant growth was analyzed by measuring rosette diameter, bolt height and chlorophyll content as a parameter of the induction of senescence.

### RNA isolation and sequencing

Total RNA was isolated using miRvana kit (Ambion) and quantified by NanoDrop. Between 1 and 3 μg of RNA from five independent plants for each treatment were pooled and sent to LC Sciences, LLC (Houston, Texas, USA) to perform the high-throughput sequencing experiments using standard Solexa/Illumina protocols. Two biological replicates for each treatment were sequenced by the Illumina platform using one independent lane for each set of replicates (two lanes total, three treatments/libraries per lane (mock-inoculated, ORMV and TMV-Cg).

### Bioinformatics analysis

Raw Data files were converted to fasta (fastq_to_fasta) and the adaptor sequence (fastx_clipper) was removed [30]. Sequences were then filtered in length (18-26nt) by an arbitrary cut off to focus in the segment with more established biological activity, and the reads were grouped and counted according to sequence identity (fastx_collapser) [30] where the header comprised of an ID and the frequency of that sequence. Only reads with more than five counts were considered for further analysis. This arbitrary cut off was made to get high levels of reproducibility since many of the sequences below that threshold are not present in both biological replicates, hence their biological relevance is questionable. These sequences reads were then mapped to *A*. *thaliana*, ORMV [GenBank:U30944.1] and TMV-Cg [GenBank:D38444] genomes using Bowtie software [31] and only the reads with perfect match were further analyzed. All counts were normalized to reads per million (RPM) according to the total read count in each library and then the average read value of unique sequences from the replicates in each treatment was used for further analysis. The sequences derived from *Arabidopsis* mapping were annotated using TAIR10 Gene Annotation Database that includes mRNA, rRNAs, tRNA, sn/snoRNAs, miRNAs, other non-coding and transposons using home-built scripts and the Galaxy web-based platform [32]. For the promoters regions, a Perl script was developed to select 500nt upstream from mRNAs that do not overlap with contiguous genes. Perl scripts were used to annotate protein coding genes into MapMan categories and associate them to their own sRNAs. Fastx_quality_stats was used for 5’-terminal nucleotide discovery [30] for all the libraries. The expected values for the viral genomes were calculated assuming that by random processing by DCL of viral RNA the 5´nucleotides would by chance be consistent with the quota of the corresponding nucleotide base in the viral sequence. R programs were used to plot the ORMV and TMV-Cg genomes. Highly structured regions (hotspots) were obtained by systematic folding of viral RNA in 150bp windows; the lowest MFE regions where then explored in detail to adjust by hand sequence length to structure. Simultaneously, viral RNA was processed with the VirMir platform [33, 34] and a ranking of premiRNA-like sequences encoded by both viruses was obtained (S1 Table). Coincident results obtained from both methods were filtered and the seven premiRNA-like sequences with higher score and lower MFE were selected for both viruses. In parallel, a selection of highly abundant vsRNA mapped to virus genomes was generated by hand, and compared with randomly selected viral sequences. The regions showing a significant higher value of mapping reads were chosen and compared with the seven premiRNA-like sequences obtained and selected (S1 Table). All scripts are available upon request.

### Northern blot hybridization

Ten μg of total RNA was used for miRNAs northern blot hybridization. Each sample was separated on 15% polyacrylamide denaturing gels and then transferred to Hybond-N+ membranes (AmershamBioScience, New Jersey, USA). The membranes were cross-linked with EDC cross-linking solution (Sigma) for 2 h at 60°C. The DNA oligonucleotides complementary to miRNAs, which were labeled with γ-32P-ATP by T4 polynucleotide kinase (New England Biolabs, Beverly, MA, USA), were used as probes for hybridization. The membranes were prehybridized with Hyb TM Plus buffer (Sigma) for 4 h followed by hybridization with the DNA probes overnight at 50°C. After washing the membranes twice with 5X SSC and once with 1X SSC and 0.1% SDS at 50°C, they were exposed for 2 days to radioactive sensitive screens, and scanned using a Thyphoon Trio scanner (AmershamBiosciences, New Jersey, USA).

### Real time quantitative polymerase chain reaction (qPCR)

For viral and mRNA detection, total RNA was treated with DNAse I (Invitrogen, California, USA). Subsequently, cDNA was synthesized using MMLV (Invitrogen, California, USA) according to manufacturer’s instructions. All qPCR experiments were carried out in an ABI StepOne Plus Real Time PCR System (Applied Biosystems, California, USA). For the experimental conditions used, following MIQE requirements, see S2 Table. The oligonucleotide primer sets used for qPCR are listed in S3 Table.

### Statistical analysis

All qPCR statistical comparisons were performed by one-way ANOVA with Tukey post-test using Infostat statistical software (InfoStat version 2008. Grupo InfoStat. FCA, Universidad Nacional de Córdoba). For all statistical analysis, significance was set as * = 0.01≤p<0.05; ** = 0.001≤p<0.01; *** = p<0.001. Contingency tables were constructed to compare the frequency distribution of the sRNAs categories between treatments; i.e. MI vs ORMV, MI vs TMV-Cg and ORMV vs TMV-CG. Statistical analyses were performed with the Pearson´s chi-square and likelihood ratio chi-square tests implemented in Infostat, (Tables 1 and 2). MapMan category obtained was compared to the Arabidopsis whole genome gene set as reference to evaluate their representation by constructing contingency tables and comparing the frequency of the different classification categories; i.e. Stress, DNA, Protein. Statistical analyses were performed with the Pearson´s chi-square and likelihood ratio chi-square tests implemented in Infostat, (Tables 3 and 4).

**Table 1:** Small RNAs reads at 2dpi mapped to *Arabidopsis thaliana* genome. Total reads were normalized to RPM. (MI) mock-inoculated; (ORMV) *Oilseed rape mosaic virus* (TMV-Cg) crucifer-infecting *Tobacco mosaic virus* infected plants.

sRNAs 2dpi	MI			TMV-Cg			ORMV		
	Unique reads	Total reads	%	Unique reads	Total reads	%	Unique reads	Total reads	%
Mature miRNAs	100	66149.0	28.1	94	38670.5	15.3	91	63550.5	24.6
TASiRNAs	73	5023.0	2.1	116	5135.0	2.0	96	8223.0	3.2
mRNAs	399	20105.5	8.5	432	28933.0	11.5	273	24570.5	9.5
pseudogenes	15	1491.5	0.6	16	680.5	0.3	16	1259.0	0.5
rRNAs	2739	79060.0	33.6	3135	107278.5	42.5	1954	103345.0	40.1
snoRNAs/snRNAs	34	232.5	0.1	25	178.5	0.1	11	122.5	0.0
tRNAs	169	18296.0	7.8	201	30614.5	12.1	148	25693.0	10.0
transposons	597	26947.5	11.5	533	20109.0	8.0	297	15676.5	6.1
promoters	794	17867.5	7.6	853	20530.5	8.1	412	15482.0	6.0
total	4920	235172.5	100.0	5405	252130.0	100.0	3298	257922.0	100.0
Statistical analysis		a≤ 0.01			b ≤ 0.01			c ≤ 0.01	

**Table 2:** Small RNAs reads at 4dpi mapped to *Arabidopsis thaliana* genome. Total reads were normalized to RPM. (MI) mock-inoculated; (ORMV) *Oilseed rape mosaic virus* (TMV-Cg) crucifer-infecting *Tobacco mosaic virus* infected plants.

sRNAs 4dpi	MI			TMV-Cg			ORMV		
	Unique reads	Total reads	%	Unique reads	Total reads	%	Unique reads	Total reads	%
Mature miRNAs	89	74303.5	21.4	158	161162.5	46.2	217	142263.5	36.1
TASiRNAs	110	3747.0	1.1	234	13326.0	3.8	465	14390.5	3.7
mRNAs	1206	32748.0	9.4	937	30931.0	8.9	2010	39597.5	10.1
pseudogenes	84	1851.5	0.5	57	1982.5	0.6	174	3345.0	0.8
rRNAs	3586	111517.0	32.1	2341	60557.0	17.4	3886	72871.0	18.5
snoRNAs/snRNAs	140	3615.0	1.0	72	924.5	0.3	232	5362.5	1.4
tRNAs	262	29887.5	8.6	178	21479.0	6.2	250	29443.5	7.5
transposons	1995	50257.0	14.5	1508	34127.0	9.8	4121	51583.5	13.1
promoters	1745	39480.5	11.4	1199	24273.0	7.0	2843	34727.5	8.8
Total	7472	347407.0	100.0	6684	348762.5	100.0	14198	393584.5	100.0
Statistical analysis		a≤ 0.01			b ≤ 0.01			c ≤ 0.01	

**Table 3:** Protein coding genes annotated to MapMan categories with their sRNAs associated at 2dpi. Total reads were normalized to RPM. (MI) mock-inoculated; (ORMV) Oilseed rape mosaic virus (TMV-Cg) crucifer-infecting Tobacco mosaic virus infected plants.

Categories (2dpi)	MI		TMV-Cg		ORMV	
MapMan	Genes	Total reads	Genes	Total reads	Genes	Total reads
Major CHO metabolism	1	14	1	66	1	42
Minor CHO metabolism	2	1062.5	2	1687	2	680.5
Lipid metabolism	2	5.5	1	5.5	0	0
Secondary metabolism	3	35.5	2	46	2	35
Hormone metabolism	0	0	1	5.5	1	37.5
Stress	8*	119	5	218.5	4	268
Misc	7	877.5	6	950.5	6	1988.5
RNA	13	2038	8	444.5	6	288.5
DNA	2*	293.5	2*	290.5	2	507.5
Protein	7*	1235	9	690.5	5	1529.5
Signalling	0	0	2	8	1	4.5
Cell	3	55	3	104	2	94
micro RNA, natural antisense etc	2	423.5	2	179.5	2	223.5
Development	5	346.5	4	187.5	4	468.5
Transport	2	30	1	167.5	1	36
Not assigned	63*	19592.5	71*	32992	54*	24612.5
Total	120	25128	120	38043	93	30816

* shows statistically different representation of each category.

**Table 4:** Protein coding genes annotated to MapMan categories with their sRNAs associated at 4dpi. Total reads were normalized to RPM. (MI) mock-inoculated; (ORMV) *Oilseed rape mosaic virus* (TMV-Cg) crucifer-infecting *Tobacco mosaic virus* infected plants.

Categories (4dpi)	MI		TMV-Cg		ORMV	
MapMan	Genes	Total reads	Genes	Total reads	Genes	Total reads
Photosynthesis	27*	1704.5	27*	3902	31*	2146
Major CHO metabolism	3	59	1	33.5	3	44.5
Minor CHO metabolism	4	1100.5	4	780	6	693
Gluconeogenesis	1	9	2	18.5	1	12
Mitochondrial electron transport	3	167.5	2	140	5	371.5
Cell wall	10	177	8	150.5	11	249.5
Lipid metabolism	6	80	4	59.5	7	75
N-metabolism	1	70.5	1	69.5	1	74.5
Amino acid metabolism	9	282	9	199.5	10	309.5
Secondary metabolism	6	271.5	5	259.5	11	358.5
Hormone metabolism	6	50	8	76.5	10	129.5
Co-factor and vitamine metabolism	1	51	1	71	1	60.5
Tetrapyrrole synthesis	0	0	1	11	3	20.5
Stress biotic	20	594.5	16*	1072	34*	1539
Redox	3	12	1	7.5	6	70.5
Polyamine metabolism	1	107	1	82	1	154
Nucleotide metabolism	1	3.5	0	0	0	0
C1-metabolism	1	22.5	0	0	2	29
Misc	26	1683.5	21	1465	35	2063.5
RNA	27	5124.5	21	2536.5	34*	3588.5
DNA	9*	1008	5*	926	11*	1187
Protein	45	1454.5	35	1973.5	55*	2662.5
Signalling	9	81.5	8	78.5	16	168
Cell	8	533.5	7	300	13	369.5
micro RNA, natural antisense etc	2	890	3	1301	3	1349.5
Development	8	160.5	6	257	10	351.5
Transport	11	186.5	6	101.5	20	223
Not assigned	172*	28484	134	29200.5	241*	41244
Total	420	44368.5	337	45071.5	581	59544

* shows statistically different representation of each category.

## Results

### Tobamovirus severity does not correlate with virus accumulation level

To investigate the involvement of a full range of sRNA species in viral infections, a comparative assay was developed using two related tobamoviruses. The comparative analysis also allowed an assessment of the role of sRNA species in both the disease process and the production of viral symptoms. The two selected tobamoviruses display some differences in symptom severity and distinctive sRNA profile along the infection process of the *A*. *thaliana* model.

For this purpose, *A*. *thaliana* Col-0 seedlings were mechanically inoculated with ORMV or TMV-Cg and mock-inoculated Col-0 plants were used as controls. Systemic leaves were sampled at two and four days post inoculation (dpi). RNA libraries were prepared and then sequenced by Illumina sequencing system (for further details refer to Material and Methods).

**Fig 1:**
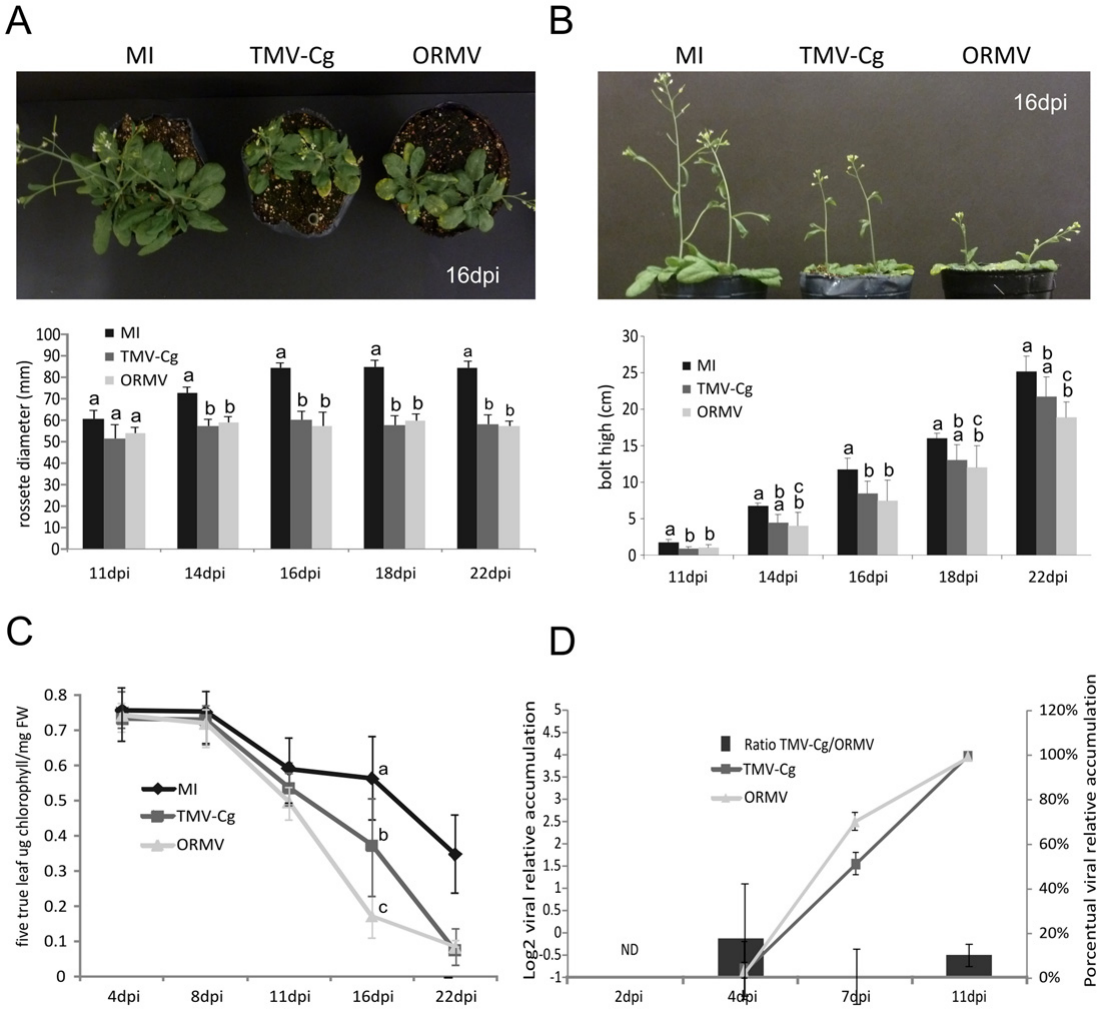
Phenotypic measurements and viral accumulation of two tobamoviruses infecting *Arabiodopsis*. (A) *Arabidopsis thaliana* Col 0 rosette at 16dpi and rosette diameter measurements of mock-inoculated (MI) and *Oilseed rape mosaic virus* (ORMV) or crucifer-infecting *Tobacco mosaic virus* (TMV-Cg) infected plants. (B) *Arabidopsis thaliana* Col0 bolt at 16dpi and bolt height measurements of mock-inoculated (MI) and TMV-Cg or ORMV infected plants. (C) Comparative progression of senescence measured as total chlorophylls content in MI, TMV-Cg and ORMV infected plants. Pigments were extracted from individual samples of only five true leaves (D) Ratio of viral loads between TMV-Cg and ORMV inoculated plants at four different dpi using tobamovirus replicase specific qPCR (Bars Graph). Relative (%) level of TMV-Cg and ORMV accumulation along the assay (Lines graph). 11dpi was arbitrary set as 100%. One-way ANOVA with Tukey’s post-test were performed. Different letters above the bars represent significant differences among groups, p-values ≤ 0.05. Error bars represent SE. * = 0.01≤p≤0.05.

Chlorophyll levels were significantly reduced at 16 dpi for both strains, being this reduction more intense in ORMV-infected plants (Fig 1C). Slighter differences were observed for bolt height at later stages of infection (Fig 1B), and no impact in rosette diameter was observed between the strains (Fig 1A), showing that while ORMV exhibit somehow an increased degree of symptom severity than TMV-Cg, the differences are mild.

Intriguingly, when the viral accumulation between viruses (Ratio represented in bars) was compared (Fig 1D), TMV-Cg showed a slightly increased accumulation than ORMV at initial stages of infection (4dpi). Later on, no differences in viral accumulation levels were found at 7 and 11dpi (Fig 1D). It is important to notice that the viral accumulation detected at 4dpi was relatively quite low, compared to the maximum level measured at 11dpi (ca. 1%) when viral accumulation was compared in a time course (Fig 1D, line graph). This finding suggests that there is no correlation between the amount of virus and symptom production.

### Sequencing and annotation of small RNAs

Two biological replicates for each treatment (mock-inoculated, ORMV and TMV-Cg) were Illumina sequenced. The raw sequence data were processed through a computational pipeline described in the Material and Methods section. Similar percentages of total sRNA sequences matched to the whole *Arabidopsis* and viral genomes in all replicates for a given treatment; which is indicative of consistency in RNA preparation and sequencing (S4 Table).

While no vsRNAs were found at 2dpi for any given library (mock-inoculated, TMV-Cg or ORMV-infected), higher abundance of vsRNAs were detected in TMV-Cg-infected plants than in ORMV at 4dpi for both replicates (approximately 13% and 8% of total sRNAs reads respectively; S4 Table). To examine the genomic distribution of the vsRNAs, a plot of normalized vsRNA read counts was generated and mapped to the viral genomes according to their polarity (S1 Fig). TMV-Cg and ORMV-derived vsRNAs mapped all along their own viral genomes and covered them nearly to saturation. The positive strand bias in vsRNA accumulation reported by other groups [26, 35] was not observed in this study. Equivalent amounts of positive and negative vsRNAs are expected from the cleavage of long dsRNA molecules generated by viral RDR during genome replication or by host RDR-mediated amplification. This possibility is consistent with the fact that at early stages there is an active viral replication. This is also in agreement to a recent study in which high-throughput sequencing analysis of viral siRNAs from ORMV-infected *Arabidopsis* did not reveal any strand bias, similar to our results, even though no data about the sampling time post-infection used is available from this work [36].

When the genomic secondary structures of both viruses were computed, only 2.2% difference in folding thermo stability was obtained, which is below to the standard error of the predictor. This finding suggests that, as a whole, both viruses share a similar overall large scale secondary folding complexity. The expected local folding of both RNA viruses was next explored and several regions were identified with a high structural complexity and low MFE (S1 Table). In parallel, local viral regions with significant higher vsRNA read counts were established. S1 Fig shows the overlapping positions of highly accumulated vsRNA read counts with highly structured regions across the viral genome (S1 Table).

### The sRNA profile of *Arabidopsis* is altered at early stages of tobamovirus infection

**Fig 2:**
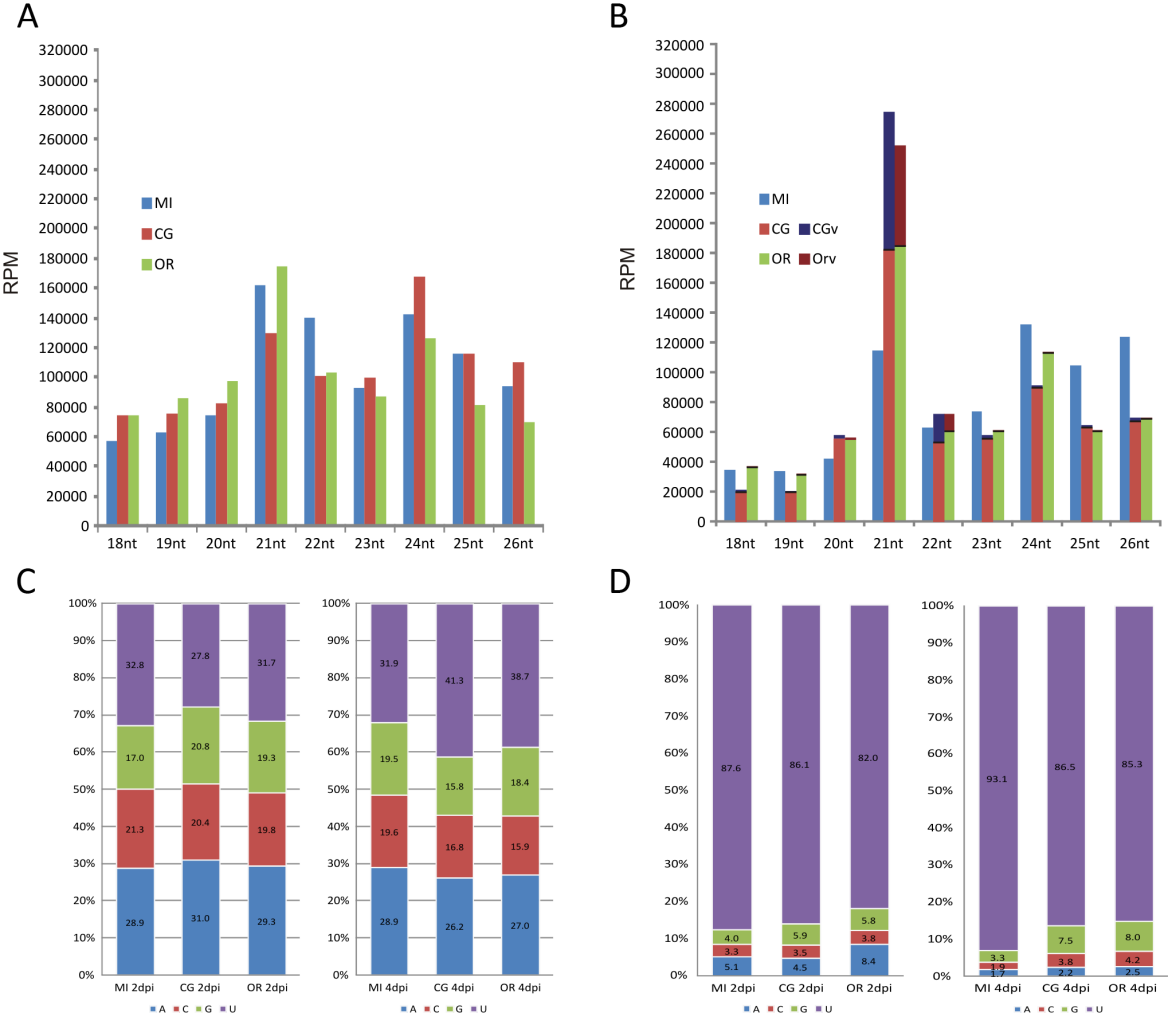
Size-specific distribution of *Arabidopsis* sRNAs and the effect of tobamovirus infection on the 5’-terminal sRNAs profile. Size-specific distribution of sRNAs in mock-inoculated (MI) and TMV-Cg or ORMV infected plants at (A) 2dpi and (B) 4dpi. VsRNAs are also shown at 4dpi. Total reads were normalized to RPM. (C) Relative frequency of each 5’ terminal nucleotide of total sRNAs compared between mock-inoculated (MI) and TMV-Cg or ORMV-infected plants. (D) Relative frequency of each 5’ terminal nucleotide of miRNAs comparing mock-inoculated (MI) and TMV-Cg or ORMV-infected plants.

The sRNA accumulation levels (reads) and percentage of sRNAs mapped to each class of annotated genomic regions between treatments at 2 and 4dpi respectively is shown in Tables 1 and 2. The global profile showed that TMV-Cg altered the proportion of sRNA accumulation at 2dpi, mostly by diminishing the shares of miRNAs and by increasing the percentage of sRNAs that mapped to the mRNA, rRNA and tRNA categories. ORMV-infected plants displayed a similar global profile as mock-inoculated plants except for the rRNA and transposon categories, which were similar to those of TMV-Cg infected plants. Interestingly, the reads that mapped to mature miRNAs were strongly reduced in TMV-Cg-treated plants in comparison to mock-inoculated plants. This phenomenon was not clearly observed in ORMV-treated plants. Such effect was accompanied by a general decline of 21nt reads, which corresponds to the most typical length of miRNAs sequences, at 2dpi in TMV-Cg plants (Table 1, Fig 2A). Interestingly, sRNAs derived from transposons accumulated to lower levels in plants treated either with ORMV or TMV-Cg (Table 1).

In contrast to 2dpi data, at 4dpi a higher accumulation of miRNA and tasiRNA reads (two folds and more than 3 fold respectively) was observed in virus-treated plants. When compared to mock-inoculated plants, sRNAs derived from transposons, promoters and mRNAs (~24nt) were diminished, particularly in TMV-Cg-infected plants, and also correlated with the size-specific distribution change of sRNAs at 4dpi (Fig 2B). The sRNAs that mapped to protein coding genes (mRNAs) showed similar levels for mock-inoculated and virus-treated plants at both sampling times. However, specific differences in the presence/absence of accumulation of several sRNA populations derived from individual genes were found. Also, a significant reduction of sRNA reads derived from rRNA was observed in virus-treated plants at 4dpi; which suggests a dynamic peculiar effect of virus infection on host rRNA. Overall, the distribution of sRNAs was significantly different between treatments by chi-square analysis (Tables 1 and 2).

At 4dpi, but not at 2dpi, the host RNA silencing machinery was also engaged in generating vsRNAs. TMV-Cg viral titers were higher than those observed for ORMV-treated plants correlating with the vsRNA levels derived from TMV-Cg and ORMV (Fig 1D and S4 Table). Fig 2B shows that the 21nt size comprises the major population of vsRNAs.

Additionally, total sRNAs according to the 5’-terminal nucleotide identity were sorted and their abundance compared among treatments and sampling times. sRNA reads with a 5’-terminal A or U were more abundant than those with a 5’-terminal C or G for all the libraries. However, while at 2dpi no differences were found among treatments, at 4dpi 5’U was overrepresented in infected plants compared to mock-inoculated controls, probably owing to miRNA enrichment in the infected samples (Fig 2C). Also, an increase of 5’G was observed within sRNA sequences at 4dpi. miRNA passenger strands (miRNA*) which are usually degraded during the biogenesis of miRNAs after the selection of the guide strand [37] were over accumulated in infected plants, and since they harbor predominantly 5´-terminal G (Fig 2D) they contributed to the increase of 5’G sRNA species.

### The accumulation of miRNAs is altered during early infection of two tobamovirus

**Fig 3:**
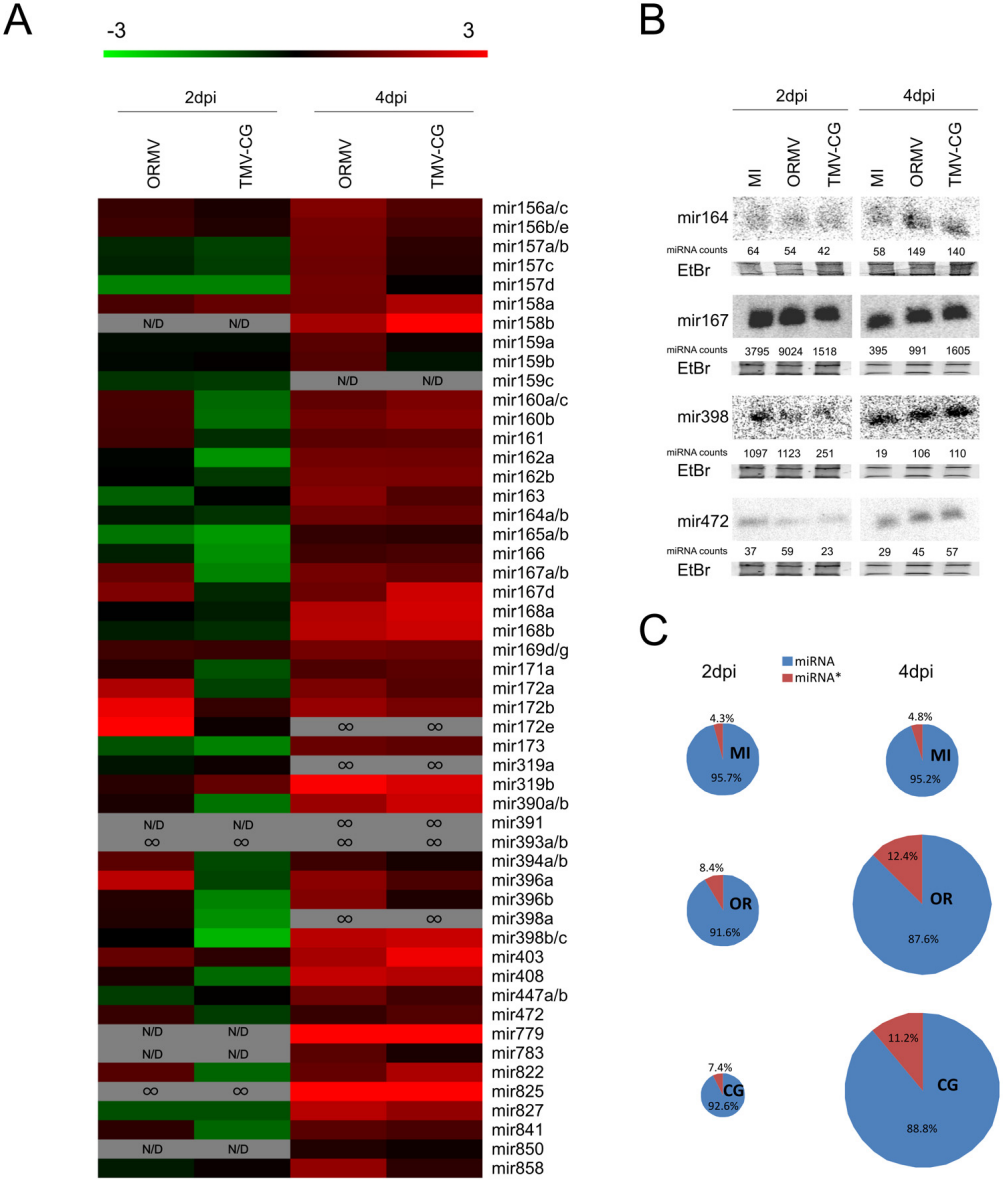
Micro RNA and miRNA* profile upon tobamovirus infection at 2 and 4dpi by high-throughput sequencing. (A) Heatmap showing log2-fold changes of average normalized miRNA reads in a double-color scale for distinct miRNAs families. Comparisons were made between mock-inoculated and TMV-Cg or ORMV-infected plants. N/D = Not detected. ∞ = Detected only in virus infected samples. For details see Supplementary S5 Table. (B) Northern blot validating the high-throughput sequencing experiments of miRNAs. Numbers are the average count reads of each miRNA family in the high-throughput sequencing experiments. (C) Effect of tobamovirus infection in the miRNA/miRNA* profile. TMV-Cg and ORMV infection produces significantly higher levels of miRNA* sequences both at 2dpi (when no virus is detected) and at 4dpi (when viral accumulation is detected). Circles size represents the miRNA amounts in each treatment.

At a global scale, the miRNA profile displayed a temporal biphasic alteration after inoculation, especially with TMV-Cg. In plants infected with this virus, a large part of the analyzed miRNAs was down regulated at 2dpi, while most of them were upregulated at 4dpi compared with mock-inoculated plants. This biphasic behavior was previously reported by our group although in a different host [38]. Moreover, previous studies using Northern blot or high-throughput sequencing indicated an enrichment of miRNAs in tobamovirus infected plants at late stages [5, 35, 39–41]. However, our global profiling was focused at very early (2dpi, no virus present) and early stages (4dpi, very low amount of virus) of infection and indicated that the phenomenon of miRNA alteration takes place earlier than reported. In a detailed analysis, some differences were observed between viruses at 2dpi. TMV-Cg infection produced a general down-accumulation of miRNAs, whereas ORMV accumulation levels differed on particular miRNAs (Fig 3A). Among the 30 miRNA families detected at 2dpi, 10 (miR160, miR161, miR167, miR171, miR172, miR390, miR394, miR396, miR398 and miR408) displayed contrasting expression levels between viruses. Indeed, most of these 10 miRNAs were down regulated by TMV-CG and upregulated by ORMV infection (Fig 3A, S5 Table). Furthermore, miR472 and miR841 showed similar patterns of accumulation, but with low read counts and less than 2 fold-change differences between treatments (S5 Table).

The validation of miRNAs expression patterns was performed by Northern blot (Fig 3B). A general correlation was observed between miRNA reads and blot signals; which reflects the reliability of the Next-generation sequencing data obtained. Slight discrepancies were observed at 2dpi in relation to miR398 and miR472 of ORMV-infected plants. The general trend, however, is consistent.

The abundance of miRNA* was assessed on infected and mock-inoculated plants. At 4dpi, viral infections caused an increased accumulation of miRNA* and probably also the associated miRNA/miRNA* duplex compared to the controls (Fig 3C). The small replicase subunit of tobamoviruses functions as a viral silencing suppressor (VSR) by binding to the sRNAs duplex. This VSR action has been proposed as one of the possible mechanisms to account for the enrichment of both miRNA and miRNA* sequences observed in infected plants [39, 42]. However, at early sampling times (2dpi), when no virus was detected in systemic leaves, an increased accumulation of miRNAs* compared to that of the corresponding miRNAs in infected plants was also observed (Fig 3C). These results cannot be explained by the action of the VSR since the virus is not present at this point. Other mechanisms, therefore, must be responsible for increasing the miRNA* population in the infected plants. For example, an alteration in the pre-miRNA processing or a modification of sRNA stabilization processes through miRNA/miRNA* duplexes methylation by HEN1 may be involved in such response. VSR might be involved in boosting this trend, which was observed at 4dpi, but our results indicate that the small replicase subunit is not directly engaged in triggering the enrichment of miRNA* species at 2dpi.

### Micro RNA targets escape pervasive miRNAs regulation during infection

**Fig 4:**
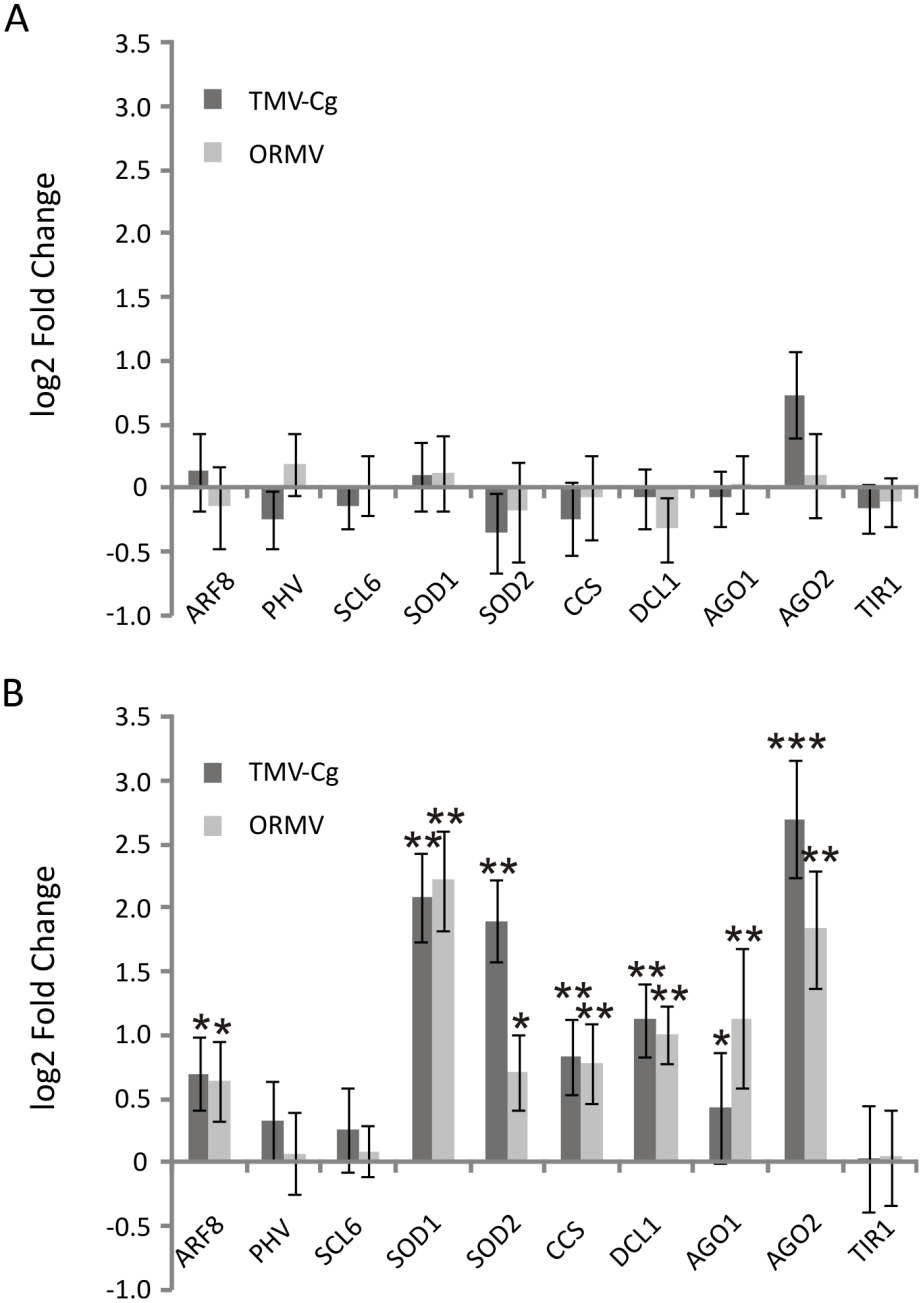
Quantitative RT-PCR analysis of selected miRNA target mRNAs upon tobamovirus infection at 2 and 4dpi. (A) Log2-fold change of miRNA target mRNA at 2dpi. Errors bars represent SE. (B) Log2-fold change of miRNA target mRNA at 4dpi. Errors bars represent SE. * = 0.01≤p≤0.05; ** = 0.001≤p≤0.01; *** = p≤0.001.

To address whether changes in the levels of miRNAs correlated to changes in their mRNA targets, a qPCR analysis of 10 well-established miRNA/mRNA targets was performed. Unexpectedly, no significant changes were observed in mRNA accumulation levels compared with mock-inoculated plants for both viruses at 2dpi (Fig 4A). The expected downregulation of the targets because of the over-accumulated miRNAs upon infection was not observed at 4dpi. Rather, most of the analyzed targets were significantly more abundant after infection with both viruses (Fig 4B). Among these targets, it can be mentioned members of the *SOD* family (targets of miR398), *ARF8* an auxin response factor (target of miR167), *DCL1* (target of miR162), *AGO1* (target of miR168) and *AGO2* (target of miR403). This positive correlation suggests an iterative feedback circle loop between mRNA target level sensing and miRNA production that may be exacerbated through a lack of function of miRNA species by virus saturation and/or impairment of the miRNA pathway [35, 38, 43]. Positive correlation might be a broader phenomenon. In a recent study focused in plant miRNAs and targets in senescing leaves, the authors’ have consistently observed a type of “incoherent regulation” (i.e. the increase in a miRNA target despite a simultaneous increase in the miRNAs which targets them) of several miRNA-miRNA-target pairs during senescence [44].

### Endogenous sRNA population targeting coding genomic regions

To analyze the impact of viral infection on sRNA populations in depth, we focused on the genomic regions that code for proteins. A significant portion of reads (10%) mapped to protein coding genes (Tables 1 and 2). All the accession numbers annotated to protein coding genes with sRNA identity were searched and the differences in their abundance among treatments were registered. Genes with functional categories and sRNA abundance were annotated by using MapMan categories and house-build scripts (S6 Table). Tables 3 and 4 grouped the genes listed into major categories with their associated sRNA read counts at 2 and 4dpi respectively. Most of the genes corresponded to non-assigned categories. Thus, Stress, RNA and Protein were the most abundant categories at both sampling times. The representation of each MapMan category was compared to the *Arabidopsis* whole genome gene set as background reference to evaluate over-representation. At 2dpi Stress category was over-represented in Mock-inoculated but not in the infected ones. Interestingly, the opposite occurs at 4dpi for the same category meaning that viral infections may play a role in the alteration of the sRNAs level that mapped to stress related genes. Interestingly, RNA was one of the categories that showed differential sRNA read counts between treatments at both dpi, and one gene accounted for most of the differences. *AT3G43990* showed decreased associated sRNAs in infected plants compared to mock-inoculated plants at both sampling times. This putative transcription regulator, which has a Bromo Adjacent Homology (BAH) domain, appears to act as a protein-protein interaction module specialized in gene silencing and is generally found in methyltransferases [45].

To discern which genes show differential sRNA levels, we selected those that displayed more than 10 read counts and 2 fold-change differences between treatments. We also retrieved the genes that had sRNA identity in only one treatment and those with differential sRNAs at the promoter regions (S7 Table). A selection of a small group of five genes was used to test by RT-qPCR whether the alteration of sRNA levels upon infection had consequences on the corresponding mRNA levels. The selection criterion narrows the list to genes related to defense, such as NBS-LRR R genes, and E3 ubiquitin ligases.

**Fig 5:**
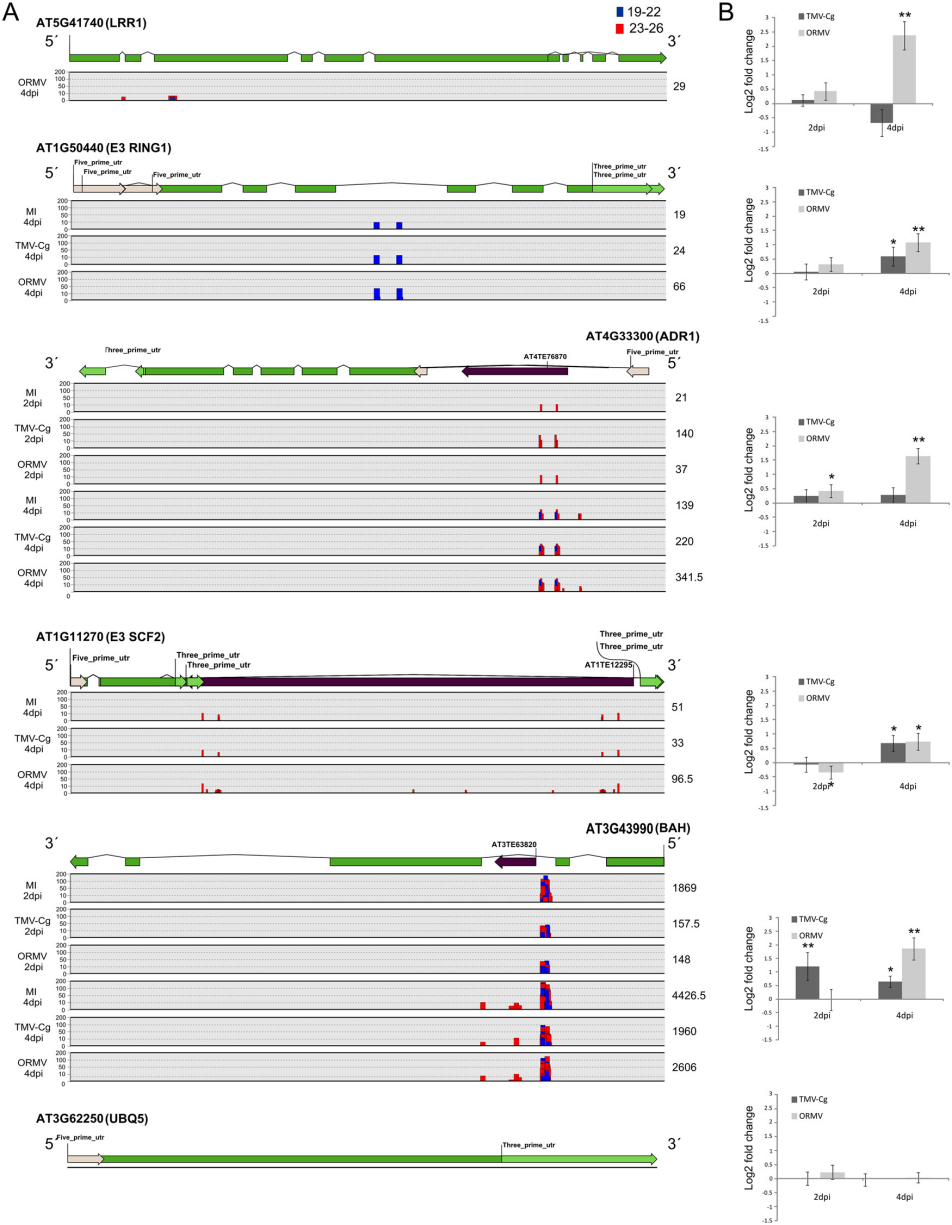
Selected genes with differential sRNA abundance. (A) Schematic representation of the position in which sRNAs mapped to the selected genes. Each bar represents the abundance and position of sRNAs mapped to the gene. Small RNAs of 19-22nt (blue bars) and 23-26nt (red bars) are represented. (B) qRT-PCR analysis of mRNAs of selected genes at 2 and 4dpi for TMV-Cg and ORMV-infected plants compared to mock inoculated. Log2 fold change of selected genes at 2 and 4dpi are shown. Errors bars represent SE. * = 0.01≤p≤0.05; ** = 0.001≤p≤0.01. AT5G41740 (LRR1) and AT4G33300 (ADR1) are members of LRRs families; AT1G50440 (E3 RING1) and AT1G11270 (E3 SCF2) are members of E3 ligases families. AT3G43990 (BAH) is a putative transcription regulator. UBQ5 gene was used as a negative control displaying no sRNAs associated and no differential transcript accumulation compared to EF1alpha housekeeping gene.

Our data revealed that sRNAs mapped at different regions of the genes and not only at the coding region. Interestingly, several genes showed sRNAs mapped at their intronic regions and at the 5' and 3' UTR regions. Some of the genes had transposable elements inserted generally at the intronic regions and some of the associated sRNAs mapped at the TE domain (Fig 5A).

At 4dpi we encountered significant differential expression in all the genes, at least in one of the viruses, concomitant with the appearance of sRNAs (Fig 5B). Furthermore, the expression level of a control gene with no associated sRNAs remained stable among treatments; which let us to think that the sRNAs that mapped in genes may have some effect on the corresponding mRNA accumulation. Particularly, a negative correlation was detected between sRNA abundance and gene expression of *AT3G43990* gene (*BAH*). The mechanisms involved in the sRNA regulation of these genes remain unclear. These mechanisms may be mediated by classical suppression via DNA methylation, this might be the case of the *AT3G43990* gene (*BAH*) regulation, or by other unknown mechanism.

## Discussion

The relationship between viral infections, endogenous sRNA accumulation and transcriptional changes associated with symptoms has been studied in depth [5, 35, 40, 41]. On the other hand, several groups have focused their studies on the impact of viral small RNAs (vsRNAs) in pathogenicity and symptom production [23–26].

Here we report a global analysis of endogenous sRNAs comparing the effects of early infections (2 and 4dpi) of two closely related tobamovirus strains (ORMV and TMV-Cg) that cause different degree of severity in *A*. *thaliana* by using high-throughput sequencing technology. The first time point was selected because of the absence of viral particles in sampled systemic tissues at this stage. This is, to the best of our knowledge, the first report analyzing sRNA accumulation at such early stages of virus infection.

### Virus-derived small RNA accumulation and their potential role in the infection processes

Firstly, we established that the slight differences in symptom severity caused by TMV-Cg and ORMV in *Arabidopsis* did not correlate with an increased viral accumulation. In fact, the virus that produces slightly milder symptoms (TMV-Cg) accumulates slightly more viral RNA than ORMV at 4dpi and this is accompanied by significantly increased vsRNA abundance (Figs 1D and 2B, S4 Table). Once the infection progresses, the viral accumulation levels increase reaching similar levels but the symptoms emerge with mild differences; which indicates that the early stages of plant-virus interaction may be critical to the symptom outcome. This observation is in agreement with the well-known fact that the level of symptomatology depends on the developmental stage of the plant at the moment of the infection; younger plants show increased symptoms than older ones in most cases [46, 47].

Moreover other groups reported variation in the vsRNA amounts depending on the virus–host system. TSWV infection in tomato and *N*. *benthamiana* resulted in a striking difference of vsRNA production between hosts (7.08% and 0.02% of total sRNAs respectively) [25], that study highlights that the phylogenetic relatedness of hosts (in this case, two related *solanaceae* species) is not a good predictor of the accumulation of virus derived sRNAs. BaMV infection in *N*. *benthamiana* and *A*. *thaliana* displayed 17.5% and 1.5% vsRNAs in systemic leaves respectively [48]. These results show that the vsRNA abundance can vary depending on both hosts and viruses, and that the sRNA outcome of viral infection in phylogenetically related hosts should be empirically tested. These variations may be attributed to a different efficiency of the RNA silencing machinery to recognize and target each viral genome. In this work we hypothesize that the correlation between vsRNAs and viral accumulation at early stages of infection is important in each virus-host combination and may be a key determinant for the progression of symptoms, even when the differences are scarce.

### Systemic changes in endogenous sRNA species at very early stages of infection

Our data indicate global changes in the endogenous sRNA species in mock-inoculated plants versus infected plants at both sampling times. A strong reduction in miRNAs from TMV-Cg infected plants but not from ORMV was observed at 2dpi (Table 1). As mentioned before, no virus was detected at that time point in sampled tissue, which could indicate that primary infection induces a rapid generation and transportation of mobile signal(s) to prepare the plant for defense or counterdefense in systemic leaves. These early differences in miRNA population accumulated after infection of the two viruses may have an impact in the differential symptoms severity.

The enrichment of the 20-21nt sRNA species, mainly from miRNA upregulation when viral infection reached systemic tissue, was previously recorded [35, 39–41]. Here we reported a 2-fold upregulation of miRNAs and 3-fold upregulation of tasiRNAs compared to mock-inoculated plants at 4dpi when low amounts of virus are present. These results indicate that the miRNA enrichment takes place unexpectedly earlier than reported and that the shift in the miRNA population from 2dpi to 4dpi occurs very rapidly (Tables 1 and 2).

Interestingly, the rRNA-derived sRNA also varied between the sampling times. Both viruses presented a mild increase at 2dpi and then an abrupt downregulation at 4dpi compared to mock-inoculated plants (Tables 1 and 2). Martinez et al (2014) described that the accumulated levels of rRNA-derived sRNAs in a Hop stunt viroid infection in cucumber correlate with an increase in the transcription of ribosomal RNA (rRNA) precursors during infection [49]. Furthermore, they also demonstrated that the deregulation of the rRNA transcripts also correlates with a dynamic modification of the DNA methylation level during the infection process. The disaggregation of rRNA-derived sRNAs in the infection was characterized by an increase in the accumulation of 21nt sRNAs (potential products of the processing of dsRNAs) and a significant decrease of 24nt sRNAs (probably involved in maintenance of methylation status) [49]. Here, even when at 4dpi the global rRNA-derived sRNAs were downregulated in infected plants, the same pattern of increase accumulation of 21nt sRNAs and decrease of 24nt compared to mock-inoculated plants was observed, especially in ORMV infected plants (Mock Inoculated: 21nt rRNA-derived = 13%; 24nt rRNA-derived = 31.7%. TMV-Cg: 21nt rRNA-derived = 22.6%; 24nt rRNA-derived = 33%. ORMV: 21nt rRNA-derived = 18.4%; 24nt rRNA-derived = 25.6%). This finding suggests an alteration of methylation in the rRNA transcripts.

### MiRNAs and miRNA targets link is not obvious during infection

It was previously reported that the alteration of several miRNAs during viral infections correlate with viral symptoms and developmental shift [5]. Interestingly, many of the miRNAs with contrasting accumulation between viruses at 2dpi have been reported to be altered by several biotic stresses, including viral infection, and the outcomes could have implications in symptom production. For instance, misregulation of *ARF8*, the gene target of miR167, could be one of the factors influencing developmental aberrations and disease symptoms by TuMV [50]. It was recently suggested, that there might be several other factors implicated in TuMV symptom development [51].

Despite differential accumulation in miRNAs between viruses at 2dpi, no changes were observed in the abundance of the analyzed mRNA targets compared to the situation in mock-inoculated plants (Fig 4A). This does not necessarily reflect that miRNAs are inactive. One plausible explanation is that in a first instance the targets may be robustly and rapidly regulated by feedback mechanisms (e.g. at the transcriptional level) [52]. Another possibility is that a translational arrest might occur, inhibiting translation of the transcripts without altering their mRNA levels. Although miRNAs in plants predominantly operate through transcript cleavage, several studies on miRNAs such as miR156, miR172, miR398, miR164 and miR165/6 show that transcript cleavage as well as translation repression may act upon the same targets [53, 54].

At 4dpi, virtually all miRNAs were upregulated for both viruses and the contrasting accumulation between them disappeared (Fig 3A; S5 Table). At this time point, some of the mRNA targets displayed changes in their transcript accumulation, being upregulated for both viruses (Fig 3B). As previously mentioned, miRNAs and its target mRNAs are usually inferred to be inversely correlated, which might be true in spatial restriction modes of regulation or the classical temporal regulation where negative correlation is evident [55]. However, in mutual exclusion modes of regulation such as miR395:*SULTR2*, both miRNA and target are transcriptionally induced upon sulfur starvation in roots [56, 57]. Moreover, miR168 and its target *AGO1* are both up-regulated after several virus infection even though the final outcome is less AGO1 protein by means of translational repression [58, 59]. Furthermore, Lopez-Gomollon et al (2012) have recently described the correlation between the expression profiles of miRNAs differentially expressed and their targets during tomato fruit development, and discovered many positively correlated microRNA/target pairs; which suggests that mutual exclusion could be as widespread as temporal regulation [43]. Positive correlation of microRNA/target pairs has been observed abundantly in plants subjected to senescing and abiotic stress [44, 60]; this “deviation” could be extended in specific cases to biotic stress. For example, Bazzini et al (2009) demonstrated that miR164 promoter respond to virus and gibberellins displaying a positive correlation of the miRNA/target pair [61]. It is important to mention that other regulation could be also acting in parallel such as transcriptional regulation of the miR/target by hormones action. Furthermore a study characterizing miRNAs promoters showed that several of them contains responsive boxes to hormones that implies that the hormones regulation is a plausible outcome in the incoherent regulation of miRNA/target pairs [62]. Finally the existence of an interference of the RISC functionality by competition with miRNAs and siRNAs pathways cannot be ruled out as suggested by Khan et al, [63] and in agreement to the recently demonstrated impact on the miRNAs level on transgenic rice plants expressing a harping construct RNAs to generate siRNAs against RSV [64]. AGO1 is proposed to be the first antiviral defense barrier while AGO2 is involved in limiting viral accumulation [65]. A plausible scenario is that at 4dpi, AGO1 might became saturated with miRNA species (loading almost strictly 5’U sRNA) preventing its onset in suppressing viral infection. This AGO1 impairment may be bypassed (or complemented) by the hierarchical action of AGO2, which loads mainly 5’A sRNA species. Interestingly, 5´A vsRNAs were slightly increased at 4dpi. This vsRNAs would be preferentially loaded into AGO2 and, thus, could be involved in limiting virus accumulation. At 4dpi Fig 4 shows a clear increase of AGO2 mRNA level supporting the idea of a complementation support for a limiting AGO1 function. Therefore it is plausible to suggest that the increased miRNAs accumulation is complemented with reduced activity due to AGO1 limitation, then the target genes increases their level. Probably ultimately inducing a positive feedback loop.

### Small RNAs that mapped to gene regions are altered during virus infection and correlate with mRNA expression

Despite the changes in the miRNA populations, mRNA target transcripts display practically no alteration between viruses that could explain the mild differences in symptom severity observed in a straight-forward manner. Therefore, in the search for differentially expressed sRNAs and more relevant correlations, we focused on endogenous sRNAs derived from genes coding for proteins (Tables 3 and 4; S6 Table). Mohorianu et al (2011) described, among others, the sRNA expression profiles associated with protein coding genes in tomato and thus identified many links to proteins with known involvement in flower and fruit development [66].

The present work discovered an extended list of sRNAs that mapped to protein coding genes with more than 2-fold differential sRNA accumulation between treatments. Among them, genes involved in defence, RNA processing and biotic stress were identified (S7 Table). Given that the sRNAs were mapped to the genomic regions with no mismatch, they may originate at these loci but also might be potential regulators in *trans* by transcriptional silencing. Although 24nt were the most abundant sRNAs mapped to gene regions, we cannot rule out post-transcriptional silencing since 21nt were also present. Nevertheless, the precise origin and function of these sRNAs is not yet fully understood. The RDR2-DCL3 pathway is involved in the generation of the most abundant 24nt sRNAs associated with genes, but in *rdr2/dlc3* backgrounds, 21nt sRNAs emerge. This finding suggests that sRNAs derived from protein coding genes are generated by several distinct pathways, including the RDR2-DCL3 [13].

As mentioned above, a few loci with sRNA differential abundance between treatments were selected to detect whether there is a link between sRNA alteration and the levels of mRNA. For instance, several E3 ubiquitin ligases were found in the list of genes, which are important in the RNA turnover and protein degradation signalling [67]; however, no significant over-representation was observed (Tables 3 and 4). Leucine Rich Repeat (LRR) domain presenting genes were also detected, which are involved in defence against pathogens [68]. *AT3G43990* (*BAH*), which is linked to RNA regulation, was selected for further analysis since it showed the most evident differential accumulation of sRNAs between different treatments.

It is interesting to mention that many of the sRNAs mapped to intronic regions. Moreover, within the five loci analysed, four displayed sRNAs matching into an intron and three showed transposable elements within those introns (Fig 5). High throughput sequencing revealed a massive amount of sRNAs derived from introns in plants [69]. Interestingly, a positive correlation between sRNAs derived from introns (i.e. sirtrons) and transcript expression of their host genes was reported; which suggests a positive self-regulation mechanism [69]. A similar correlation for some of the loci evaluated and, more importantly, differential expression between viruses was found in this study. One hypothesis is that the production of sRNAs from the introns depends on the level of expression of their host genes, hence the positive correlation. This finding suggests that these sRNAs may have an impact in the emergence of symptoms besides the known influences of miRNAs at latter stages of infection. How these sRNAs work is not apparent but it is likely that they would be capable of regulating their region of origin in *cis* or yet other related genes in *trans*, like the multigenic family of LRRs [70]. Additionally, it is important to mention that three out of the five putative target genes showed differential expression levels between both viral infections (Fig 5B).

As previously mentioned, *AT3G43990* (*BAH*) displayed the major differences in sRNA accumulation in the RNA MapMan category among treatments, especially when comparisons were made between comparing infected plants and mock-inoculated controls (Fig 5A). The information regarding this locus is scarce except for the presence of a BAH domain. Recent works described two genes in which the BAH domain along with the RNA Recognition Motif (RRM) domain facilitates appropriate processing of transcripts that have large intronic regions associated with TE [71, 72]. *AT3G43990* (*BAH*) does not have the RRM domain at the 3’ end found in the other genes that seems to be necessary to mask the intronic region post-transcriptionally and to allow the production of the full-length mRNAs [71, 72]. However, this gene did have a putative TFIIS domain that stimulates RNA pol II to transcribe through regions of DNA that promote the formation of stalled ternary complexes [73]. Differential expression of *AT3G43990* (*BAH*) mediated by sRNAs upon viral infection could have an impact in the correct transcription of some genes. However, more studies are necessary to assess how *AT3G43990* (*BAH*) could affect others transcripts.

To the best of our knowledge, this work represents the initial step in uncovering how differential accumulation of endogenous sRNAs could contribute to the massive alteration of the transcriptome associated with plant-virus interactions. Consequently host-derived sRNAs may have a crucial role in the production of differential symptoms in plants at very early stage of tobamovirus infection.

## Supporting Information

S1 Fig Distribution of viral small RNAs through tobamovirus genome. TMV-Cg vsRNAs mapped to TMV-Cg viral genome in either sense or antisense orientation (left panel) and ORMV vsRNAs mapped to ORMV viral genome in either sense or antisense configuration (right panel). The abundance of vsRNAs was calculated and plotted as the sum of normalized reads in a 20 nucleotide sliding window along the viral genome. Highly structural regions (hotspots) across the viral genome are shown.
(TIF)

S1 Table Summary of the viral highly structured regions and viral small RNAs analysis for TMV-Cg and ORMV infection at 4dpi. Description of data: The footnotes of the table are as follows: *viral hotspots*: Outline of the hotspots obtained for TMV-Cg and ORMV. *TMV-Cg MFE and ORMV MFE*: Systematic folding of viral RNA in ~150bp windows. The structured regions selected in the TMV-Cg and ORMV genome are highlighted in green. *VirMir TMV-Cg*: ranking of the best premirRNA-like sequences encoded in the TMV-Cg genome. *VirMir ORMV*: ranking of the best premirRNA-like sequences encoded in the ORMV genome. *TMV-Cg hotspots structure*: folding structure of the 7 hotspots selected for TMV-Cg genome. *ORMV hotspots structure*: folding structure of the 7 hotspots selected for ORMV genome. *Mapping hot TMV-Cg >100 count*: TMV-Cg local viral regions with more than 100 count reads of vsRNAs mapped to the hotspots. The main vsRNAs are shown. *Mapping hot ORMV >100 count*: ORMV local viral regions with more than 100 count reads of vsRNAs mapped to the hotspots. The main vsRNAs are shown.
(XLS)

S2 Table MIQE requirements for qPCR analysis. (DOCX)

S3 Table Oligonucleotide primers used for qPCR experiments. (DOCX)

S4 Table Number of total mappable RNA reads and mapped sRNA reads to *Arabidopsis thaliana* and viral genomes (TMV-Cg and ORMV) for both replicates. (TIF)

S5 Table Normalized average reads of miRNAs and miRNAs* in mock-inoculated, TMV-Cg and ORMV infected plants at 2 and 4dpi. The footnotes of the Table are as follows: *miRNA 2dpi*: normalized count reads of mock-inoculated (MI), TMV-Cg and ORMV of miRNA and CG/MI; OR/MI ratio in fold change and log2-fold change of miRNA at 4dpi. *Micro RNA 4dpi*: normalized count reads and CG/MI; OR/MI ratio in fold change and log2-fold change at 2dpi. *Micro RNA* * *2dpi*: normalized count reads of MI, TMV-Cg and ORMV miRNA* at 2dpi. miRNA* 4dpi: normalized count reads of MI, TMV-Cg and ORMV miRNA* at 4dpi.
(XLS)

S6 Table Protein coding genes annotated with functional categories and normalized associated sRNA abundance. The footnotes of the table correspond to the different treatments and sampling times.
(XLSX)

S7 Table Protein coding genes displaying more than 10 reads counts and 2 fold-change differences among treatments. The footnotes of the table correspond to the different treatments, relations and sampling times.
(XLS)
