# Supplementary figures and images for: Differential mRNA Accumulation upon Early Arabidopsis thaliana Infection with ORMV and TMV-Cg Is Associated with Distinct Endogenous Small RNAs Level
Source: PLoS One. 2015 Aug 3;10(8):e0134719. doi: 10.1371/journal.pone.0134719 (PMC4597857; doi:10.1371/journal.pone.0134719)

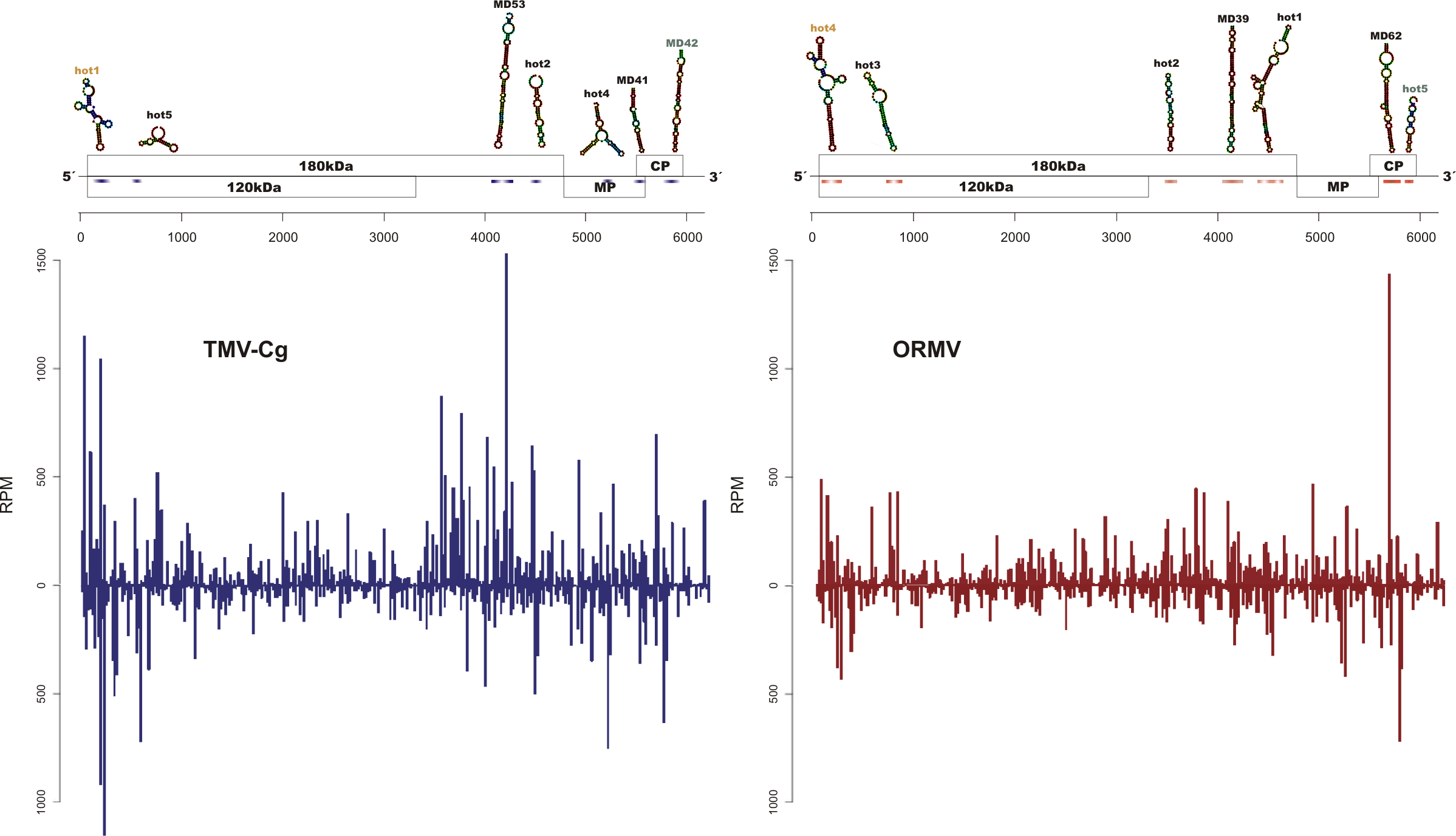

Supplement: S1 Fig — TMV-Cg vsRNAs mapped to TMV-Cg viral genome in either sense or antisense orientation (left panel) and ORMV vsRNAs mapped to ORMV viral genome in either sense or antisense configuration (right panel). The abundance of vsRNAs was calculated and plotted as the sum of normalized reads in a 20 nucleotide sliding window along the viral genome. Highly structural regions (hotspots) across the viral genome are shown. (TIF) [file pone.0134719.s001.tif]

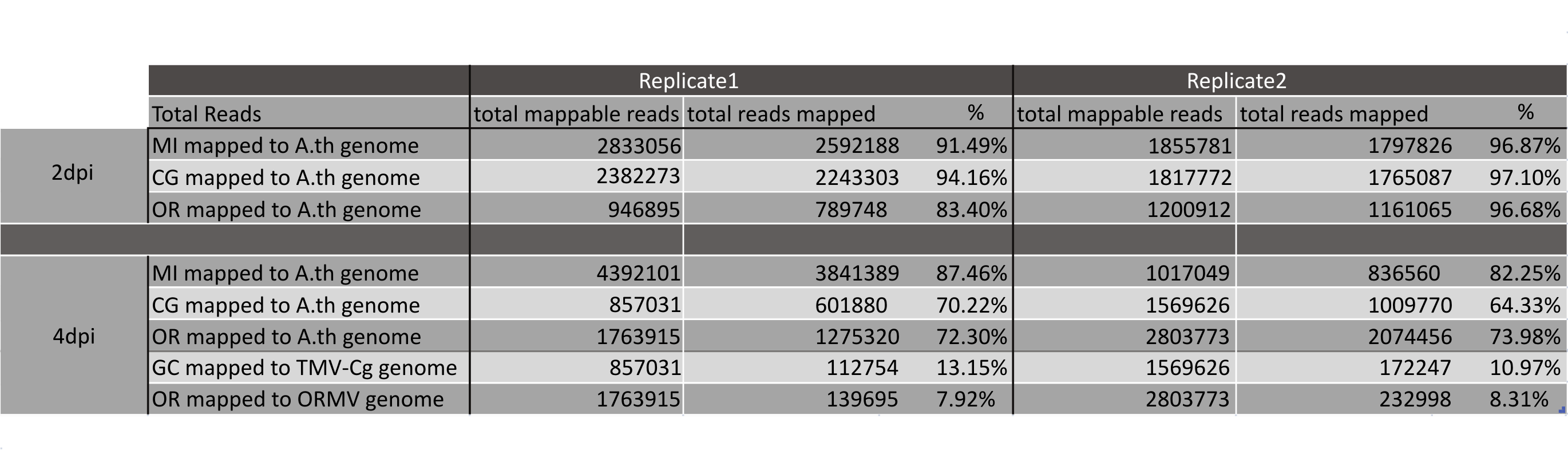

Supplement: S4 Table — (TIF) [file pone.0134719.s005.tif]
