# Supplementary material for: Differential mRNA Accumulation upon Early Arabidopsis thaliana Infection with ORMV and TMV-Cg Is Associated with Distinct Endogenous Small RNAs Level
Source: PLoS One. 2015 Aug 3;10(8):e0134719. doi: 10.1371/journal.pone.0134719 (PMC4597857; doi:10.1371/journal.pone.0134719)
Supplement: S2 Table — (DOCX) [file pone.0134719.s003.docx]

| **Experimental design** |  |
| --- | --- |
| Control groups | *A.thaliana* Col0 buffer-inoculated |
| Treatment groups | *A.thaliana* Col0 inoculated with either TMV-Cg or ORMV viruses |
| **Sample** |  |
| Type of sample | 6^th^ and 8 ^th^ leaves (2dpi) / 8 ^th^  and 11 ^th^ leaves (4dpi) |
| Processing procedure | Liquid nitrogen homogenization |
| Sample frozen conditions | -80 ºC |
| Biological replicates | n=4-8 for each treatment and time point |
| **RNA extraction** |  |
| Procedure | Acid Phenol extraction |
| Reagents | TRIzol (Invitrogen) |
| Details of Dnase treatment | DNAse I Amp Grade (Invitrogen), 15 min at room temperature |
| Contamination assessment | < 3% |
| Nucleic acid quantification | Absorbance at 260 nm |
| Instrument and method | NanoDrop instrument |
| Purity( A260/ A 280) | > 1.8 |
| RNA integrity | Analyzed by agarose gel electrophoresis |
| **Reverse transcription** |  |
| Complete reaction conditions | Reaction was performed as described by the manufacturer´s instructions. |
| Amount of RNA and reaction volume | 1 µg of RNA, 20 µl |
| Priming oligonucleotide | Random primers (Invitrogen) |
| Reverse transcriptase | M-MLV (Invitrogen) |
| Temp and time | 10 min 50 ºC, 50 min 37 ºC, 15 min 70 ºC. |
| **qPCR protocol** |  |
| Complete reaction conditions (*) | 5 min 95 ºC, (15 s 95 ºC, 30 s 60 ºC, 40 s 72 ºC) x 45 cycles |
| Reaction volume and amount of cDNA | 2 µl of a 1/20 dilution of synthesized cDNA in a final volume of reaction of 20 µl. |
| Primers, Mg^2+^ and dNTPs concentration | 3 mM Mg^2+^, 200 nM primers, 0,2 mM dNTPs |
| Polymerase | Platinum® Taq DNA Polymerase (Invitrogen) |
| Buffer | 20 mM Tris-HCL (pH = 8.4), 50 mM KCl |
| Manufacturer of qPCR instrument | Step One Plus, Applied Biosystems |
| **qPCR validation** |  |
| Specificity | Analysed by agarose gel and Melting Curve parameters on each qPCR run. |
| Method of PCR efficiency calculation | Mean PCR efficiency per amplicon calculated by LingRegPCR program (Ramakers *et al*. 2003). |
| **Data analysis** |  |
| qPCR analysis program | LinRegPCR program |
| Method of Cq determination | LinRegPCR program |
| Outlier identification | LinRegPCR program |
| Justification of number and choice of reference genes | 3 reference genes tested, TUB4 (NM_123801), UBQ5 (NM_116090) and EF1-α (NM_125432)) for stability using the Bestkeeper stability algorithm. EF1- α was chosen as the most stable reference gene. |
| Description of normalization methods | (Pfaffl *et al*. 2002):  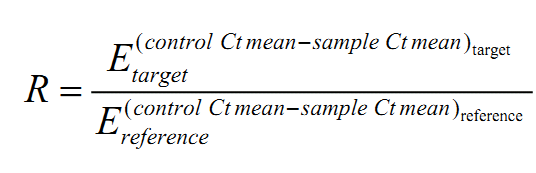 |
| Number of technical replicates | 2 |
| Statistical method | Permutation test |
| Software | fgStatistics software (Di Rienzo J.A, 2009, (<http://sites.google.com/site/fgStatistics/>) |
| Repeatability (intraassay variation) C_q_ SD error | Between 0.05 and 0.20 depending on the assayed amplicon. |
