# Supplementary material for: Differential mRNA Accumulation upon Early Arabidopsis thaliana Infection with ORMV and TMV-Cg Is Associated with Distinct Endogenous Small RNAs Level
Source: PLoS One. 2015 Aug 3;10(8):e0134719. doi: 10.1371/journal.pone.0134719 (PMC4597857; doi:10.1371/journal.pone.0134719)
Supplement: S3 Table — (DOCX) [file pone.0134719.s004.docx]

| **AGI identifier** | **Description** | **Fwd Primer (5'-3')** | **Rev Primer (5'-3')** |
| --- | --- | --- | --- |
| D38444 | Replicase TMV-Cg/ORMV | TGTCGCAATCGTATCAAAC | CTGTATCTGGAAACCGCTG |
| AT1G19680 | RING | AGTTTTCGACGGGACAACAGGAGG | CCCAGTTTCAGATGACGGAGGTCC |
| AT3G23900 | RRM | AAGGAGAGAAAGACGCTGCGGC | TCAGATTCCGGTGCGGATTCC |
| AT2G07050 | CAS1 | TCTTGGGGGAAAATGTGGCTATCG | CGACAATGGCACCACATCCTTCC |
| AT1G54370 | NHX5 | GCATTGGAGCTTCATCTGACGAGG | CTCCCCATCTCCATCTCCATCTCC |
| AT3G26590 | MATE | GTCGTTGCCGGCTTCTCATTCG | CCATGATCGTTGCAAGTAAACGCC |
| AT1G54920 | UNK | TCCAACATCATCCAATGTGTTGGC | GAGCGTGAAAGCGTCAACTTCGTT |
| AT1G75710 | C2H2 | AGCTTCATCAAGCAGTTCGTCACG | TTCACTGCGTCTCGGCAATCC |
| AT5G37020 | ARF8 | AGATGTTTGCTATCGAAGGGTTGTTG | CCATGGGTCATCACCAAGGAGAAG |
| AT1G30490 | PHV | AGACCTTGGCGGAGTTCCTTTG | GTTGCGTGAAACAGCTACGATACC |
| AT4G00150 | SCL6 | ACCAAGACCAGTCAGCGGTAATC | AGTGTCGTCGTTGTTGTTGTTAAGG |
| AT1G08830 | SOD1 | CCAAAGAGAGACGAAGCA | GCCTTTGTGATCTCAGGA |
| AT2G28190 | SOD2 | GCTCCAGAAGATGAGTGCCGTCA | CCACCCTTTCCGAGGTCATCCTT |
| AT1G12520 | CCS | GATCTCACAAACGGAGCAGCCAGCA | TTCTAGCAATCACTGCGGCGGTCAA |
| AT1G01040 | DCL1 | GATCCATTCCTAAGCGAAGTTTCAGAG | GCCCGAGCAACATAAAGATCCATAG |
| AT1G48410 | AGO1 | AAGGAGGTCGAGGAGGGTATGG | CAAATTGCTGAGCCAGAACAGTAGG |
| AT1G31280 | AGO2 | CCCCAATAACGCAGTTTTA | CAAATTCGTTTCAACACACC |
| AT3G62980 | TIR1 | GCCTCTCTCTATCTGGCCTCTTGAC | AGGGCAGCTCTCTGGTCTCGAGTCC |
| AT5G41740 | LRR1 | TGTGATGACGAGTGGAGTGTTCGG | TCCCTCGACGGTGTGAGATTCAGT |
| AT1G50440 | E3 RING1 | TCGAATTTGCCTTGATGTTGGAGG | CCATCTATCAGCAGGCACATTGGC |
| AT4G33300 | ADR1 | ACAGAAGAAAGCGGTTGCTGATGC | TCTGCCTTGGGGAACTCCATGTC |
| AT1G11270 | E3 SCF2 | TGGATCAGTTCATTGGCTCACCG | CCATATCAGTTGGGCAGGACCG |
| AT3G43990 | BAH | AACCCGATCTCGGAGAAGTT | TCACGATCCAAAGCACTGGATGG |
| AT5G60390 | EF1alpha | ATTGGTAACGGTTACGCCCC | TCTCCTTACCAGAACGCCTGTC |
| AT3G62250 | UBQ5 | CGGACCAGCAGCGATTGATT | ACGGAGGACGAGATGAAGCG |
| AT5G44340 | TUB4 | CTGTTTCCGTACCCTCAAGC | AGGGAAACGAAGACAGCAAG |
